# Data-driven energy landscape reveals critical genes in cancer progression

**DOI:** 10.1038/s41540-024-00354-4

**Published:** 2024-03-08

**Authors:** Juntan Liu, Chunhe Li

**Affiliations:** 1 Institute of Science and Technology for Brain-Inspired Intelligence, Fudan University, Shanghai, 200433 China; 2 Shanghai Center for Mathematical Sciences, Fudan University, Shanghai, 200433 China; 3 School of Mathematical Sciences and MOE Frontiers Center for Brain Science, Fudan University, Shanghai, 200433 China

**Keywords:** Multistability, Cancer

## Abstract

The evolution of cancer is a complex process characterized by stable states and transitions among them. Studying the dynamic evolution of cancer and revealing the mechanisms of cancer progression based on experimental data is an important topic. In this study, we aim to employ a data-driven energy landscape approach to analyze the dynamic evolution of cancer. We take Kidney renal clear cell carcinoma (KIRC) as an example. From the energy landscape, we introduce two quantitative indicators (transition probability and barrier height) to study critical shifts in KIRC cancer evolution, including cancer onset and progression, and identify critical genes involved in these transitions. Our results successfully identify crucial genes that either promote or inhibit these transition processes in KIRC. We also conduct a comprehensive biological function analysis on these genes, validating the accuracy and reliability of our predictions. This work has implications for discovering new biomarkers, drug targets, and cancer treatment strategies in KIRC.

## Introduction

The evolution of a biological system can be conceptualized as a dynamic and nonlinear system that changes over time^1–3^. Throughout the process of evolution, multiple stable states are often established, and transitions occur between these states^4^. Especially, for cancer research, it is crucial to correctly identify the stable states and characterize the evolution process based on experimental data, since the transition between different cell states in cancer systems plays a pivotal role in the evolution of cancer, such as the progression of cancer cells through epithelial-mesenchymal transition (EMT)^5–9^. Therefore, it is of great importance to effectively identify stable states and corresponding transitions of cancer systems, as well as to unveil the critical genes involved in these intricate processes.

Currently, cancer is one of the leading causes of death worldwide^10^. It can occur in various organs and tissues of the human body, including but not limited to the lungs, breasts, colon, prostate, and cervix. The development of cancer typically involves abnormal cell proliferation and differentiation, leading to the formation of malignant tumors^11^. To address the challenges posed by cancer, extensive research has been conducted globally with the aim of improving early detection rates^12^, developing more effective treatment methods^13^, and implementing prevention strategies^14^. However, cancer remains a complex and challenging problem that requires interdisciplinary collaboration and sustained efforts to overcome its challenges.

Experimental studies play a vital role in investigating the biological processes involved in cancer development, progression, and treatment response^15^. Although experimental methods are essential, they can be costly and influenced by various unstable factors. Advancements in sequencing technologies have paved the way for data-driven approaches in cancer research. This approach enables quantitative investigation of cancer evolution, identification of stable states, and discovery of relevant genes. Understanding the mechanisms of cancer progression through quantitative methods is critical, as it can provide valuable insights into the precise characterization of disease evolution.

To study cancer dynamics, an important question is to identify the stable state in the system and corresponding transitions between stable states. For example, unsupervised learning techniques have been used to study the transition dynamics of epithelial-to-mesenchymal transition using single-cell transcriptomic data^6^. Also, the gene-gene regulatory relationships have been investigated to identify system states and their associated transition genes^5^. Additionally, the model-based energy landscape methods are employed to study system evolution and quantify the transition path, along with the exploration of gene regulatory networks^4,16,17^. So, data-driven energy landscape may provide an effective approach to study the evolution mechanism of cancer.

In this study, we employed a data-driven energy landscape algorithm^18^ to analyze the progression of cancer and investigated its significant transitions, such as onset and deterioration. Using Kidney renal clear cell carcinoma (KIRC) cancer as an example, we analyzed its evolutionary process. KIRC is a common and deadly form of cancer. It is resistant to conventional treatments^19^, heterogeneous^20^, and lacks curative options for metastatic cases. To understand the molecular mechanisms underlying KIRC, we analyzed data from The Cancer Genome Atlas (TCGA) project. This large-scale sequencing dataset provides unprecedented opportunities to uncover new insights into cancer development.

We propose two novel indicators, based on transition probability and barrier height (Supplementary Figure 1) to identify crucial promoting or inhibitory genes in the transition process. Furthermore, we discover that these genes are associated with important pathways or relevant biological processes related to cancer. For instance, in the onset of KIRC cancer, dopaminergic synapses^21^, and the *cAMP* signaling pathway^22^ were found participating in this transition. In the deterioration of KIRC cancer, it was observed that the Neuroactive ligand-receptor interaction, p53 signaling pathway^23^, cell cycle^24^, and cAMP signaling pathway^22^ were enriched with a significant number of genes.

We conducted a detailed analysis of two transition processes and identified the critical genes using both indicators. Specifically, we observed that these genes are primarily enriched in cancer-related processes. For instance, during the transition from stage TA to I, *KRT4* and *MMP3* were identified as critical genes. The expression of *KRT4* and *KRT17* can be used to determine whether an individual has cervical cancer^25^. Additionally, the expression of the *MMP3* gene in keratinocytes promotes differentiation and inhibits tumor formation^26^. During the transition from stage III to IV, the critical genes identified were *CALCA* and *NR0B2*. Methylation of *MGMT* and *CALCA* could be used as new molecular markers of prognosis in testicular germ cell tumors (TGCT)^27^. Furthermore, the orphan nuclear receptor NR0B2 may represent a new susceptibility locus associated with early-onset colorectal cancer^28^.

## Results

### Data-driven energy landscape reveals KIRC disease progression

**Fig. 1:**
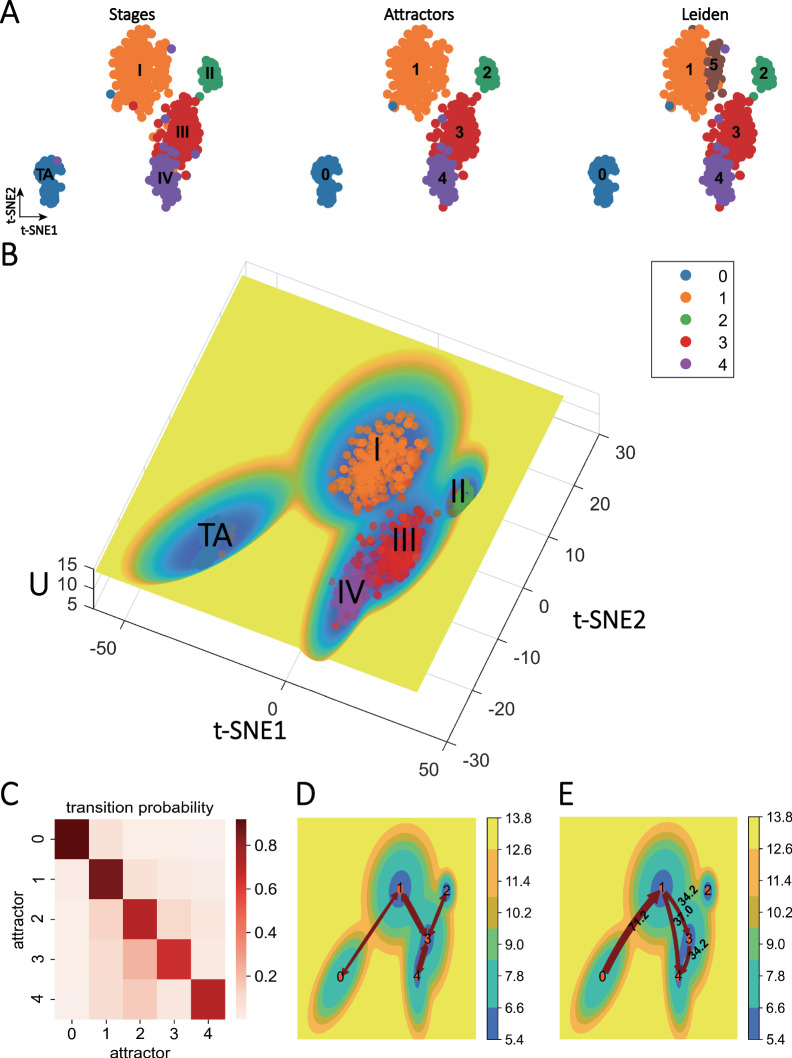
Data-driven landscape reveals KIRC disease progression. **A** Left: Tumor stage labels of samples under t-SNE dimensionality reduction, which is divided according to tumor diameter size, where TA refers to tumor-adjacent samples; Middle: MuTrans classification results by attractors of the dynamical system; Right: Unsupervised clustering algorithm Leiden based on gene expression data analysis of the population results. It can be seen that the attractor has a general correspondence with the staging label (Supplementary Figure 2), while the samples of stage I (attractor 1) in the Leiden cluster tend to be divided into two categories: group 1 and group 5. **B** The three-dimensional energy landscape corresponding to the KIRC data shows the results of different stages (attractors): the x-axis is t-SNE1, the y-axis is t-SNE2, and the ordinate is the energy magnitude (see formula 8), where there is a general correspondence between the staging information and the attractor, and each attractor corresponds to a stable state. **C** Transition probability matrix between each attractor: the color on the diagonal is darker and the other parts are lighter, indicating that each attractor is relatively stable. **D** The trajectory inference of the MPFT method: results were demonstrated in the two-dimensional energy plane. The color shade represents the energy value, the arrow line indicates that there is a transition path between the two attractors, and the obvious paths 0-1-3-4,2-3-4 can be seen in the figure. **E** The trajectory inferred by the MPPT method: the arrows from attractor A to B indicate that A transits to B. There are two significant paths for 0- > 1- > 3- > 4, and 0- > 1- > 4, where attractor 2 seems to be isolated.

KIRC cancer is a common and deadly cancer, and studying its evolutionary mechanisms and proposing potential therapeutic strategies is a crucial question. Now, with advances in sequencing technology, it is essential to provide novel insights into cancer research based on experimental data. By applying a data-driven energy landscape method (MuTrans)^18^, we analyzed the KIRC cancer data to study its evolution (Fig. 1A). The KIRC dataset contained tumor stage labels of patients (based on tumor size), which were divided into 5 categories, i.e., tumor-adjacent (TA), stages I, II, III, and IV (Supplementary Table 1). We used the Eigen-Peak Index (EPI) strategy (Supplementary Note 1) to detect the number of attractors in the data, with the number of attractors matching the number of labels (Supplementary Figure 2). The attractor detection results obtained using the MuTrans correspond well to the patients’ staging information (Supplementary Figure 2), indicating that each stage of patients can be roughly characterized by the corresponding attractor (middle panel of Fig. 1A). According to the clustering results of the unsupervised learning algorithm Leiden, each stage corresponded to a group while samples in stage I (attractor 1) tend to be divided into two groups: group 1 and group 5 (right panel of Fig. 1A). Furthermore, we constructed a three-dimensional energy landscape to visualize the results of different stages (attractors), where each basin represents an attractor (or a stable state), and the color depth represents the energy value. From Fig. 1B, it can be observed that each stable state can be well characterized.

Next, we aim to quantify the transition between different attractors (stable states). We calculated the transition probability matrix between attractors (Fig. 1C). As shown in the figure, the values on the diagonal were significantly higher, indicating that the cells in the corresponding attractor have the greatest probability of transition back to the original attractor, so the corresponding attractor behaves more stable (i.e., reaching a stable state). However, there are also noticeable transitions between different attractors, such as transition between attractors 2 and 3. Furthermore, we applied trajectory inference algorithms Most Probable Path Tree (MPPT) and Maximum Probability Flow Tree (MPFT) to analyze the evolutionary trajectory^18^. In the MPFT graph (Fig. 1D), we observed two distinct paths: 0- > 1- > 3- > 4 and 2- > 3- > 4. Among them, attractor 3 (stage III) is a critical stable state, which is at the central node position. There are two obvious transition pathways passing through attractor 3, which may indicate that it is an intermediate state of KIRC cancer evolution, and the cells in attractor 3 may return to a benign state (stage I, II) with appropriate intervention and treatment, or rapidly deteriorate to stage IV, if without timely treatment. Therefore, there is an opportunity to identify corresponding warning signals in stage III^29^.

With the MPPT method (Fig. 1E), we provide the initial point (attractor 0; stage TA) and the endpoint (attractor 4; stage IV). The results shown in the two-dimensional energy plane indicate that the transition follows the path 0- > 1- > 3- > 4 and 0- > 1- > 4. Particularly, attractor 2 (stage II) seems to be isolated and not on the evolutionary trajectory, and this stable state may also be an important intermediate state of KIRC, which is more difficult to evolve into a malignant state. From the transition streamlines, it can be inferred that attractor 1, as a bifurcation point, may have significant implications, as many cases rapidly deteriorate from stage I to stage IV. Combining both methods, it is hypothesized that the path 0- > 1- > 3- > 4 represents a consistent differentiation pathway. Considering the clinical significance, we found that attractor 0- > 1 (stage TA- > I) and attractor 3- > 4 (stage III- > IV) correspond to cancer onset and cancer progression, which is of great significance.

### Identification of critical genes for transition from stage TA to I

In the above analysis, we discovered the main evolutionary pathway of KIRC disease to be 0- > 1- > 3- > 4. During this process, the onset and progression of the disease are crucial. In this section, we investigated the onset process of KIRC and proposed two quantitative indicators, based on transition probability and barrier height to identify the critical genes that play a critical role in the onset of cancer. Furthermore, we conducted a biological functional analysis to further explain the biological processes involved.

**Fig. 2:**
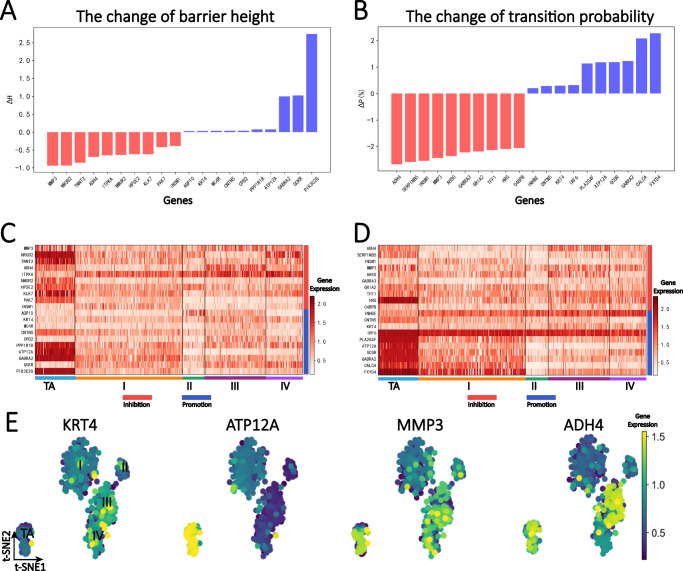
Critical gene analysis of the onset period (from stage TA to I). **A** The barrier height indicator identified the top ten promoting and inhibitory genes in cancer onset: among which the energy barrier height from TA to I was 4.6. $\varDelta H$ greater than 0 (blue column) represents corresponding gene promoting the cancer onset process, and $\varDelta H$ less than 0 (red column) represents corresponding gene inhibiting the cancer onset process (see Methods for detailed definition of $\varDelta H$). **B** The transition probability indicator detected the top ten promoting and inhibiting genes in cancer onset, in which the transition probability value was 81.3% when all gene expression information was included in the process from stage TA to I (see Methods for details). **C** The gene expression heatmap of the top ten promoting and inhibitory genes detected by barrier height indicator. **D** The gene expression heatmap of the top ten promoting and inhibitory genes detected by transition probability indicator. **E** Distribution of gene expression data with shared critical genes identified by the two indicators. Darker blue indicates lower expression, while darker yellow indicates higher expression. The expression values of *KRT4* gene were relatively uniformly distributed. *ATP12A* was differentially expressed in TA samples but significantly reduced in other samples, which may be related to the pathogenesis. *MMP3* showed significantly high expression in stages TA, III, and IV, while *ADH4* was differentially expressed in stages TA and III.

According to Fig. 2A, we can observe that different genes have different effects on the energy barrier height. We used simulated gene knockout to study the effect of gene expression on barrier height. The change of barrier height caused by the gene was defined as the barrier height difference before and after the gene knockout (see Method). The expression of some genes decreases the energy barrier height, while others increase it. From biological perspective, this corresponds to promoting and inhibiting the onset of diseases. The degree of each gene’s effect can be quantified from the change in the energy barrier height. Similarly, from Fig. 2B, we can also see that the effect of genes can be indicated by the change in the transition probability. Therefore, it can be inferred that when the transition probability increases (decreases) due to gene expression change, it promotes (inhibits) the process.

We also identified the shared critical genes identified by the two indicators (Fig. 2E), and found that for these shared critical genes, the magnitude of our two indicators can also reflect their functional importance. For example, both *KRT4* and *ATP12A* are among the top 10 genes identified by both indicators as factors of promoting cancer onset. However, *ATP12A* is significantly higher than *KRT4* in both indicators (ranking high, Fig. 2A, B), and from the gene expression distribution (Fig. 2E), *ATP12A* shows significantly higher expression in TA samples, while *KRT4* does not exhibit significant differential expression. As for the genes *MMP3* and *ADH4*, which are identified by both indicators as inhibitory factors for cancer onset, they are both in the top 4 positions of both indicators (Fig. 2A, B), indicating a strong inhibitory role in the KIRC cancer onset process. Moreover, they both show significantly higher expression in TA samples. For the *ADH4* gene, it also shows significantly higher expression in stage III samples, so may also participate in some critical process in stage III, which confirms that stage III is a critical period (consistent with the trajectory inference results in Fig. 1).

Moreover, as depicted in Fig. 2C and D, it is evident that several genes crucial in the onset of KIRC are differentially expressed genes (DEGs). Notably, the *NROB2* and *ATP12A* genes exhibit significantly higher expression levels in the TA samples. Conversely, genes such as *KRT4* (*p_adj* = 0.38) and *MC4R* (*p_adj* = 0.98) do not display substantial upregulation in specific stages of the samples (see Supplementary Table 2 and Supplementary file for detailed DEG analysis results). Consequently, our approach differs from the conventional method of gene selection based on differential expression analysis. Our method possesses the potential to identify genes that are not differentially expressed, often disregarded in the initial screening using traditional approaches, yet playing a pivotal role in specific biological processes. This capability is advantageous for the discovery of novel biomarkers, drug targets, and innovative strategies for cancer treatment.

### Identification of critical genes for transition from stage III to IV

In the previous section, we analyzed the onset process of KIRC cancer and identified the corresponding critical genes. We also found that in clinical practice, we mainly deal with patients who have already developed cancer, and most of these patients are diagnosed at a late stage due to the lack of significant early symptoms and early treatment. Therefore, studying how cancer patients transition from benign tumors to malignant tumors is also an important topic. In recent years, there have been studies on constructing early warning signals for critical stages of diseases from data-driven or model driven approaches^30–33^.

**Fig. 3:**
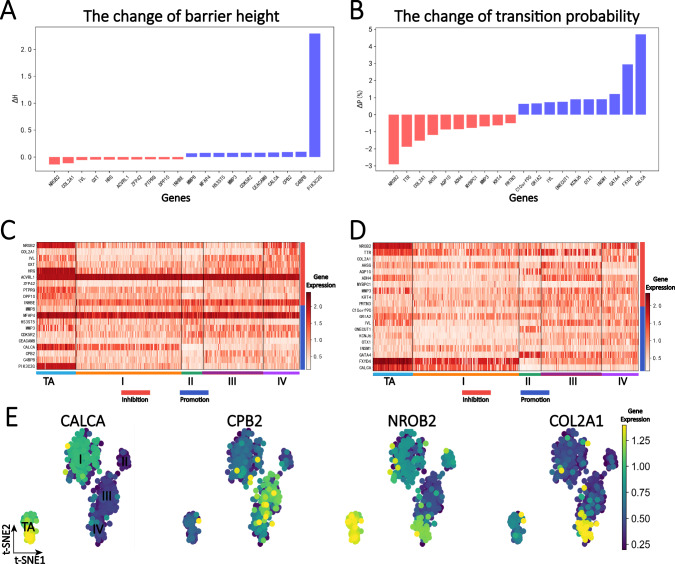
Critical gene analysis of the progression period (from stage III to IV). **A** The barrier height indicator identified the top ten promoting and inhibitory genes in cancer progression, among which the energy barrier height from III to IV was 0.3. $\varDelta H$ greater than 0 (blue column) represents corresponding gene promoting the cancer progression, and $\varDelta H$ less than 0 (red column) represents corresponding gene inhibiting the cancer progression (see Methods for detailed definition of $\varDelta H$). **B** The transition probability indicator detected the top ten promoting and inhibiting genes in cancer progression, in which the transition probability value was 69.5% when all gene expression information was included in the process from stage III to IV. **C** The gene expression heatmap of the top ten promoting and inhibitory genes detected by barrier height indicator. **D** The gene expression heatmap of the top promoting and inhibitory genes detected by transition probability indicator. **E** Distribution of gene expression data with shared critical genes identified by the two indicators. Darker blue indicates lower expression, while darker yellow indicates higher expression. *CALCA* was differentially expressed in stages TA and I samples, *CPB2* was differentially expressed in stage III samples, *NROB2* was differentially expressed in stages TA and IV, and *COL2A1* was differentially expressed in stage IV.

In this section, we primarily investigated the progression of KIRC cancer from stage III to stage IV. We proposed two indicators to detect critical genes involved in this process, as shown in Fig. 3A and B. The critical genes in the figure were sorted by their effect size, and their gene expression heatmaps were displayed in Fig. 3C and D. Some genes, such as *NROB2*, *HRG*, *PIK3C2G*, and *GATA4*, are differentially expressed genes (DEGs), while some genes such as *COL2A1*, *CPB2*, *AQP10*, *KCNJ6* are non-differentially expressed genes that were often overlooked. Additionally, genes like *ACVRL1* and *MFAP4* exhibited high expression in all stages of the samples compared to other genes, suggesting their involvement in more complex interactions.

Combined with Fig. 3E, *CALCA* and *CPB2* were identified as genes that promote the deterioration process. *CALCA* was differentially expressed in stages TA and I samples, and with lower expression in other stages, which may indicate that *CALCA* is involved in some complex regulatory mechanisms and affects cancer exacerbation^27^. *CPB2* gene was differentially expressed in stages III and IV, which means that its expression may directly participate in cancer deterioration^34^. In addition, *NROB2* and *COL2A1* were identified as inhibition of KIRC deterioration. *NROB2* was differentially expressed in TA, which may indicate the expression of *NROB2* will inhibit the tumor deterioration, while *COL2A1* was differentially expressed in IV, indicating that the expression of this gene may involve some other regulatory mechanisms to inhibit tumor deterioration. These two genes ranked in the top three in the ordering from the two indicators, and the expression level was more significant than other genes, indicating that they were relatively active in KIRC tumor cells^28,35^.

We also compared the impact of different normalization methods on barrier height and transition probability (Supplementary Figs. 3 and 4). We found that using the normalization method from Method 1 (utilized in our work), the attractors identified corresponded well to the stage of the sample, while other normalization methods did not achieve a good correspondence, resulting in biases in the calculated values of barrier height and transition probability. Therefore, appropriate normalization methods are essential for better associating attractors with stages of cancer, further characterizing the evolution of cancer. Furthermore, we also conducted an exploration of the potential correlation between barrier height and transition probability, and we observed that both indicators exhibited a certain degree of linear relationship (Supplementary Fig. 5).

### Functional analysis of critical genes

We have investigated the critical genes of different transition processes in a data-driven manner. However, the specific biological significance of these results still needs to be further validated through existing biological experiments and enrichment analysis. In this section, we performed Gene Ontology (GO) and Kyoto Encyclopedia of Genes and Genomes (KEGG) functional enrichment analysis on the identified genes by the two indicators to further elucidate their involvement in cancer mechanisms, providing a reference for future research on KIRC cancer.

**Fig. 4:**
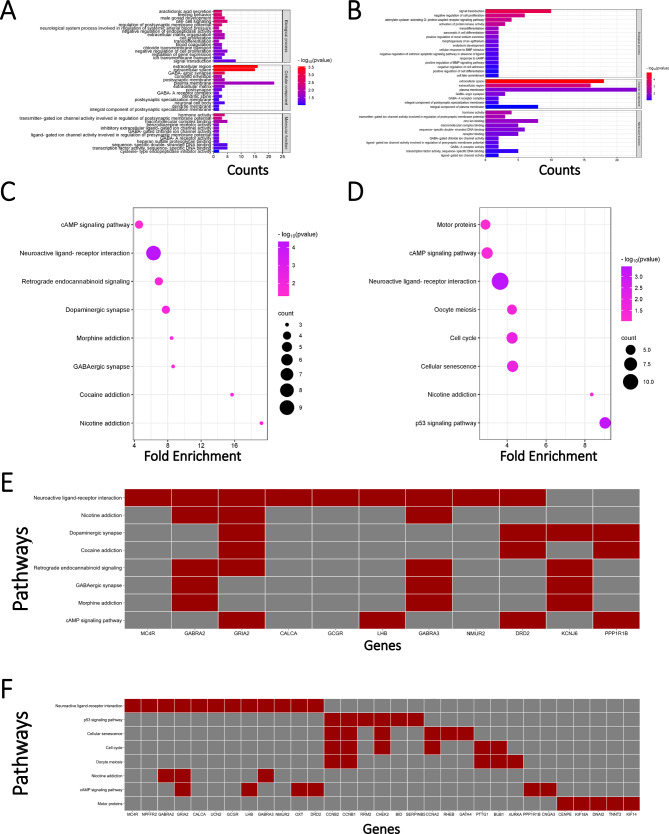
Functional analysis of critical genes in the KIRC onset and progression period. **A** GO enrichment analysis of critical genes identified in the KIRC cancer onset: The enrichment results of the top 20 promoting and inhibitory genes from both indicators were analyzed in terms of Biological Process, Cellular Component, and Molecular Function. **B** GO enrichment analysis of critical genes identified in the KIRC cancer progression: The enrichment results of the top 20 promoting and inhibitory genes from both indicators were analyzed in terms of Biological Process, Cellular Component, and Molecular Function. KEGG pathway enrichment analysis of critical genes (**C**) KIRC cancer onset and (**D**) KIRC cancer progression: The x-axis represents Fold Enrichment, the color intensity represents the significance of pathway enrichment, and the size of the circles represents the number of genes enriched. Mapping of specific critical genes in KEGG pathway enrichment (**E**) KIRC cancer onset and (**F**) KIRC cancer progression: Red (gray) indicates the specific critical genes that are enriched (not enriched) in this pathway.

As shown in Fig. 4A and B, we performed GO functional analysis on critical genes identified for the process of cancer onset and progression, individually. The analysis was conducted in three dimensions: Biological process, Cellular component, and Molecular function. It was observed that these genes are enriched in many biological processes and functions related to cancer, such as cell proliferation^36^, and plasma membrane^37^. Additionally, we conducted a KEGG pathway analysis on these genes. In the process of cancer onset (Fig. 4C), we found that these pathways, such as the p53 signaling pathway^23^, are related to the mechanisms of cancer development. On the other hand, pathways such as nicotine addiction are associated with specific individual behaviors and previous studies have shown that behaviors like smoking greatly increase the risk of cancer^38^. In the progression of the disease (Fig. 4D), these genes are involved in pathways, such as the cell cycle^24^, and the p53 signaling pathway^23^, closely related to cancer. These results further demonstrate the effectiveness of our model predictions.

In Fig. 4E and F, we provided a detailed display of the specific genes enriched in these pathways. In the onset of KIRC cancer, it was observed that the Neuroactive ligand-receptor interaction, Dopaminergic synapse^21^, and the *cAMP* signaling pathway^22^ are enriched with a significant number of genes. Furthermore, genes such as *GABRA2*, *GRIA2*, *GABRA3*, *DRD2*, and *KCNJ6* are found to be enriched in these pathways. In the deterioration of KIRC cancer, it was observed that the Neuroactive ligand-receptor interaction, p53 signaling pathway^23^, cell cycle^24^, and the *cAMP* signaling pathway^22^ are enriched with a significant number of genes. Additionally, genes such as *CCNB2*, *CCNB1*, and *CHEK2* are found to be enriched in these pathways. In conclusion, our proposed method effectively identifies critical genes that play a crucial role in KIRC cancer either for cancer onset or disease progression.

In the subsequent analysis, we conducted a detailed investigation of each transition process and identified the critical genes associated with each process, as summarized in Table 1. Specifically, we found that the *KRT4* gene plays a promoting role in the TA- > I transition process. Furthermore, the expression of both *KRT4* and *KRT17* can serve as indicators for the presence of cervical cancer in individuals^25^. On the other hand, *MMP3* was identified to have an inhibitory role in this transition process. Notably, the expression of *MMP3* in keratinocytes enhances differentiation and effectively prevents the establishment of tumors^26^. Moving on to the transition from stage I to stage II, we discovered that the *CPB2* gene plays a promoting role^34^, while the *INSM1* gene has an inhibitory effect^39^. In the subsequent transition from stage II to stage III, both the *GATA4* and *AQP10* genes were identified to have promoting roles^40,41^. In the transition from stage I to stage III, the *COL2A1* gene was identified to have a promoting role^35^, while the *GRIA2* gene was identified to have an inhibitory role^42^. Finally, in the transition from stage III to stage IV, the *CALCA* gene was identified to have a promoting role^27^, while the *NR0B2* gene was identified to have an inhibitory role^28^. The relation between these critical genes identified by the two indicators and cancer was demonstrated in Table 1.

**Table 1:** The critical genes identified by the two indicators in the five transitions

Transition	Gene	Type	Category	Relation with cancer progression
TA- > I	*KRT4*	Promotion	Structural protein	Expression of *KRT4* and *KRT17* can identify whether a subject has cervical cancer^25^
	*MMP3*	Inhibition	Enzyme	Keratinocyte expression of *MMP3* enhances differentiation and prevents tumor establishment^26^
I- > II	*CPB2*	Promotion	Enzyme	*CPB2* toxin may lead to abnormal cell apoptosis and functions in porcine small intestinal epithelial cells^34^
	*INSM1*	Inhibition	Transcription factor	*INSM1* gene expression could be used to predict neuroendocrine tumor histology^39^
I- > III	*COL2A1*	Promotion	Structural protein	Expression profile of *COL2A1* and the pseudogene *SLC6A10P* predicts tumor recurrence in high-grade serous ovarian cancer^35^
	*GRIA2*	Inhibition	Transport channel	*GRIA2* is a novel diagnostic marker for solitary fibrous tumor identified through gene expression profiling^42^
II- > III	*GATA4*	Promotion	Transcription factor	A *GATA4*-regulated secretory program suppresses tumors through recruitment of cytotoxic CD8 T cells^40^
	*AQP10*	Promotion	Transmembrane water channel proteins	*AQPs* have important roles in cancer cell growth, migration, invasion, and angiogenesis, each of which is important in human carcinogenesis^41^
III- > IV	*CALCA*	Promotion	Signaling hormone	Methylation of *MGMT* and *CALCA* could be used as new molecular markers of prognosis in testicular germ cell tumors (TGCT)^27^
	*NR0B2*	Inhibition	Enzyme	The orphan nuclear receptor NR0B2 may represent a new susceptibility locus associated with early-onset colorectal cancer^28^

## Discussion

How to effectively quantify the dynamic processes of biological systems using mathematical methods is an important issue. Nonlinear dynamical system theory is commonly used to model biological systems, where the stable states of the system can be represented by attractors of the dynamical system. However, with the explosion of biological experimental data and the characteristics of real data such as noise, large samples, and high dimensionality, it has become quite difficult and urgent to construct interpretable models based on data. For cancer research, it is also paramount to develop data-driven approaches to study the mechanism of cancer dynamics, and correctly identify the stable states and measure the evolution process.

In this work, we applied a data-driven energy landscape method to learn the dynamical characteristics of nonlinear dynamical systems. With KIRC as an example, we effectively identified the stable states (attractors) of the system and trajectory inference methods (MPFT, MPPT) were used to infer the transition trajectories between stable states and identify essential transition paths. Additionally, we utilized the energy landscape method to quantify the dynamic transition process for KIRC, where the points with lower energy (higher probability density function) are more stable.

Furthermore, for each transition process, we proposed two indicators to study the specific effect of each gene on the transition in terms of energy barrier height and transition probability, determining whether it promotes or inhibits the transition. In the disease progression, we found that the *NROB2* and *ATP12A* genes were significantly upregulated in stage TA samples, suggesting their potential involvement in the pathogenesis. On the other hand, genes such as *KRT4* and *MC4R* did not show significant upregulation in certain stages of the samples^25^. In the study of KIRC cancer progression, *CALCA* and *CPB2* were identified as genes that promote the deterioration process, and *CALCA* showed significant expression in stage TA and I samples, followed by a decrease in expression. This may indicate the involvement of *CALCA* in complex regulatory mechanisms that affect cancer progression. However, from the perspective of gene expression, we found that the genes we identified are partially differentially expressed.

Genes identified with uniform distribution may interact with other genes, but these interactions may vary across different attractors. In more detail, when calculating the similarity matrix at the sample-sample level, while the expression of genes is relatively uniform, the magnitude of its effect on each attractor may vary due to differences in the number of samples in each attractor, resulting in changes in barrier height and transition probability. The mechanism of these critical gene’s role in KIRC cancer is more complex. How to study these genes in more detail based on data and gene regulatory networks is one of our future research directions. In addition, we found that not only these critical genes identified by our indicators play a significant role in the evolution of KIRC cancer but also some of them are non-DEGs, often discarded by most researchers in the initial screening stage. Therefore, it has the potential to find new biomarkers, drug targets, and new cancer treatment strategies.

In further analysis, we performed biological functional analysis on the selected critical genes. In the onset of KIRC cancer, it was observed that the Neuroactive ligand-receptor interaction, Dopaminergic synapse^21^, and the *cAMP* signaling pathway^22^ were enriched with a significant number of genes. Furthermore, genes such as *GABRA2*, *GRIA2*, *GABRA3*, *DRD2*, and *KCNJ6* were found to be enriched in these pathways. In the deterioration of KIRC cancer, it was observed that the Neuroactive ligand-receptor interaction, p53 signaling pathway^23^, cell cycle^24^, and the *cAMP* signaling pathway^22^ were enriched with a significant number of genes. Additionally, genes such as *CCNB2*, *CCNB1*, and *CHEK2* were found to be enriched in these pathways.

Our work involves determining the transition paths and investigating the role of genes in each specific transition. However, the sequential expression of genes or the effect of genes may change the barrier height and transition probability, which may lead to changes in the selection of transition paths. For instance, when the expression of a gene increases the barrier height of a certain transition process or decreases the transition probability of that process, it implies that the evolution of cancer may be more inclined to avoid that process and choose an alternative path. Of course, this also requires us to compare the barrier height and transition probability of this transition path with those of other transition paths, which is an interesting and meaningful research direction and will be a focus of our future work.

In summary, we have applied a data-driven energy landscape approach to study KIRC cancer. We have proposed two strategies to investigate two important transition processes of cancer, identified critical genes, and conducted corresponding biological functional analyses for their biological significance. Our research contributes to understanding the evolutionary process of KIRC cancer and the identification of critical genes involved in this process. This provides valuable insights for clinicians and scientists in finding new biomarkers, drug targets, and novel cancer treatment strategies.

## Methods

### Theoretical background

Cellular evolution can often be modeled as a dynamical system using stochastic differential equations (SDEs), as follows:

1 $$d{X}_{t}=f({X}_{t}){dt}+\sigma \left({X}_{t}\right)d{W}_{t}$$

where ${{\rm X}}_{t}\in {{\mathbb{R}}}^{p}$ is the gene expression value of the cell at the moment $t$, $f({X}_{t})$ is the drive force containing the interaction relationship between genes, etc., $\sigma ({X}_{t})$ is the noise size of the system, and ${W}_{t}$ is the standard Brownian motion. When the number of genes is small, $f({X}_{t})$ can be estimated using causal inference algorithms^43^. However, when the number of genes is too large (typically exceeding the limit in single-cell sequencing), direct fitting or solving of high-dimensional Eq. (1) is not feasible. In such cases, a multiscale data-driven method is employed to reconstruct the structure of the dynamical system, where each steady state of the system is represented as an attractor. This approach is further described in the following section.

### The workflow of the MuTrans algorithm

The Mutrans algorithm aims to uncover the dynamics underlying sequencing data by considering three key aspects^18^: 1) Computing the random-walk transition probability matrix (rwTPM) at the cell-cell level. 2) It focuses on identifying the attractors of the nonlinear dynamical system, classifying each cell accordingly, and computing the rwTPM at the cluster-cluster level. Then lineage inference approach can be applied to infer the transition paths between attractors (categories). 3) Computing the rwTPM at the cell-cluster level. By using membership probability, we can denote the probability of the cell belonging to the attractor. By addressing these perspectives, the Mutrans algorithm provides valuable insights into the dynamics of the sequencing data.

### Computing the rwTPM at the cell–cell level

The transition probability matrix, measured at the cell–cell level using the random walk model, can be directly calculated from the gene expression data. It is defined as follows:

2 $$p\left(x,y\right)=\frac{w\left(x,y\right)}{d\left(x\right)},d\left(x\right)=\sum _{z}w\left(x,z\right)$$

In this context, $x,y$ represents the cell, and $w(x,y)$ represents the distance between cells $x$ and $y$. The distance metric used can be Euclidean distance, cosine similarity, correlation coefficient, or any other appropriate measure. Under this definition, the stationary probability distribution of the transition probability matrix is denoted as $\mu (x)=\frac{d(x)}{\sum _{z}d(z)}$, and it satisfies the detailed-balance condition $\mu (x)p(x,y)=\mu (y)p(y,x)$.

### Computing the rwTPM at the cluster–cluster level

In this step, the number of attractors needs to be determined. This can be achieved by employing the EPI strategy (Supplementary Note 1; Supplementary Figure 2) to assess the gene expression data. Alternatively, if the label information of cells or marker genes of cells is known, prior knowledge about the number of label categories can be used to determine the number of attractors.

The transition probability matrix $\bar{{\rm P}}={({\bar{P}}_{{ij}})}_{K\times K}$ is first defined at the cluster-cluster level, where ${\bar{P}}_{{ij}}$ represents the probability of attractor ${S}_{i}$ transiting to ${S}_{j}$, $K$ represents the number of attractors, and based on the cluster-cluster random walk transition probability matrix (rwTPM) $\bar{{\rm P}}={({\bar{P}}_{{ij}})}_{K\times K}$, the cell-cell rwTPM is further constructed as follows:

3 $$\bar{p}\left(x,y\right)=\sum _{i,j}{1}_{{S}_{i}}\left(x\right){\bar{P}}_{{ij}}{1}_{{S}_{j}}\left(y\right)\frac{\mu \left(y\right)}{{\bar{\mu }}_{j}}$$

where ${\bar{\mu }}_{j}=\sum _{y}{1}_{{S}_{j}}(y)\mu (y)$, and ${1}_{{S}_{j}}(z)$ is the indicator function, which means that if the cell $Z$ belongs to attractor ${S}_{j}$, then ${1}_{{S}_{j}}\left(z\right)=1$, otherwise ${1}_{{S}_{j}}\left(z\right)=0$.

For cluster-cluster rwTPM $\bar{{\rm P}}={({\bar{P}}_{{ij}})}_{K\times K}$ and the attractor clustering result, we can calculate them using the optimization method as follows:

4 $$\mathop{\min }\limits_{{S}_{K},{\hat{P}}_{{ij}}}{{\big\Vert}\bar{p}\left[{S}_{K},{\bar{P}}_{{ij}}\right]-p{\big\Vert}}_{\mu }^{2}$$

where $\mu$ is the stationary probability distribution of the cell-cell rwTPM $p$, ${{\rm{||}}A{\rm{||}}}_{\mu }^{2}=\sum _{x,y}\frac{\mu (x)}{\mu (y)}A{(x,y)}^{2}$, and this optimization problem can be solved iteratively^18^. The optimized ${{S}_{K}}^{* },{{\bar{P}}_{{ij}}}^{* }$ obtained in this study measure the stability of the system and its inter-transition characteristics using a probabilistic model. Additionally, the Most Probable Path Tree (MPPT) approach or Maximum Probability Flow Tree (MPFT) approach can be applied to infer the transition paths between attractors.

### Computing the rwTPM at the cell-cluster level

Constructing cell-cluster rwTPM by introducing membership function $q(x)=({q}_{1}(x),{q}_{2}(x),..,{q}_{K}(x))$, where ${q}_{i}\left(x\right)$ represents the probability that cell $x$ belongs to attractor ${S}_{i}$, and $\sum _{i}{q}_{i}(x)=1$. Based on $\bar{{\rm P}}={({\bar{P}}_{{ij}})}_{K\times K}$ obtained from the previous step, the cell-cell rwTPM can be constructed as follows:

5 $$\widetilde{p}\left(x,y\right)=\sum _{i,j}{q}_{i}\left(x\right){\bar{P}}_{{ij}}{q}_{j}\left(y\right)\frac{\mu \left(y\right)}{{\widetilde{\mu }}_{j}},{\widetilde{\mu }}_{j}=\sum _{x}{q}_{j}\left(x\right)\mu \left(x\right)$$

The solution for $q\left(x\right)$ can be obtained by the following optimization problem,

6 $$\mathop{\min }\limits_{q}{{\rm{||}}\widetilde{p}\left[q\right]-p{\rm{||}}}_{\mu }^{2}$$

$$s.t.\,q(x)=({q}_{1}(x),...,{q}_{K}(x)),\mathop{\sum }\limits_{i=1}^{K}{q}_{i}(x)=1$$

where $\bar{{\rm P}}={({\bar{P}}_{{ij}})}_{K\times K}$ is optimized when constructing the cluster-cluster rwTPM, this problem can be solved by the quasi-Newton method^18^.

### Inferring the transition path

In order to further quantify the evolution of the system, that is, the transition paths between different attractors, combined with the ${{S}_{K}}^{* },{{\hat{P}}_{{ij}}}^{* }$ obtained by the Mutrans method, the transition probability matrix $\hat{P}={({\hat{P}}_{{ij}})}_{K\times K}$ is first defined at the cluster scale, where ${\hat{P}}_{{ij}}$ represents the probability of attractor ${S}_{i}$ transferring to ${S}_{j}$, $K$ represents the number of attractors. We infer the trajectory based on the transition path theory using two methods, namely the Most Probable Path Tree (MPPT) approach and the Maximum Probability Flow Tree (MPFT) approach.

#### The maximum probability flow tree (MPFT)

The proposed method utilizes the concept of system evolution, which typically exhibits a tree-like structure. By incorporating the theory of minimum spanning trees, a trajectory graph is constructed to optimize the flow of maximum transition probabilities. The cluster-cluster transition probability matrix ${{\hat{P}}_{{ij}}}^{* }$ satisfies the detailed-balance condition ${\mu }_{i}^{* }{{\hat{P}}_{{ij}}}^{* }={\mu }_{j}^{* }{{\hat{P}}_{{ji}}}^{* }$. Therefore, we can construct a symmetric probability flow matrix $F$, where ${F}_{{ij}}={\mu }_{i}^{* }{{\hat{P}}_{{ij}}}^{* }$. Here, the flow ${F}_{{ij}}$ represents the percentage of cells transitioning from attractor ${{S}_{i}}^{* }$ to ${{S}_{j}}^{* }$ relative to all cells undergoing transitions in the Markov chain. Using Kruskal’s algorithm to construct a maximum spanning tree of an undirected graph from a matrix, such that the generated tree has the maximum probability flow of transitions^18^.

#### The most probable path tree (MPPT)

In contrast to MPFT, MPPT can determine specific paths and their probabilities between attractors, given the initial and final states. This capability is valuable for studying differentiation processes. Given the initial state ${{S}_{i}}^{* }$ and the end state ${{S}_{j}}^{* }$, for all possible paths connecting ${{S}_{i}}^{* }$ and ${{S}_{j}}^{* }$, the relative likelihood of each transition path, defined as the sum of the capacity of all paths from ${{S}_{i}}^{* }$ to ${{S}_{j}}^{* }$ on its path capacity ratio, can be interpreted as the proportion of effective transition fluxes along the developmental trajectory of interest, which can be constructed in graph theory as the shortest path tree^44–47^.

Of course, other pseudo-time ordering methods such as PAGA^48^, DPT^49^, Slingshot^50^ and Monocle^51^ etc. can also be used for analysis.

### Construction of energy landscape

After determining each attractor, we apply a Gaussian distribution fitting to each attractor in the two-dimensional space (using dimensionality reduction methods such as PCA, t-SNE, etc.). Specifically, for attractor ${S}_{i}$, we fit its Gaussian distribution $N({\vec{\mu }}_{i},{\sum }_{i}),$ ${\hat{\vec{\mu }}}_{i}=\frac{1}{{N}_{i}}\mathop{\sum }\nolimits_{j=1}^{{N}_{i}}{\vec{X}}_{j}$, ${\hat{\sum }}_{i}=\frac{1}{{N}_{i}}\mathop{\sum }\nolimits_{j=1}^{{N}_{i}}({\vec{X}}_{j}-{\mu }_{i}){({\vec{X}}_{j}-{\mu }_{i})}^{T}$, where${\vec{X}}_{j}$ denotes the coordinates of cell j in two-dimensional space in the attractor ${S}_{i}$, ${N}_{i}$ denotes the total number of cells in attractor ${S}_{i}$.

7 $${P}_{{S}_{i}}\left(\vec{X}\right)=\frac{1}{\sqrt{2\pi \left|{\hat{\sum }}_{i}\right|}}{e}^{-\frac{{\left(\vec{X}-{\hat{\vec{\mu }}}_{i}\right)}^{T}{{\hat{\sum }}_{i}}^{-1}\left(\vec{X}-{\hat{\vec{\mu }}}_{i}\right)}{2}}$$

Gaussian mixture model (GMM) fitting yields the total probability density function, i.e.

8 $$P\left(\vec{X}\right)=\mathop{\sum }\limits_{i=1}^{K}{w}_{i}{P}_{{S}_{i}}\left(\vec{X}\right)$$

And its energy value $U(\vec{X})=-{InP}(\vec{X})$, ${w}_{i}=\frac{{N}_{i}}{\mathop{\sum }\limits_{i=1}^{K}{N}_{i}}$, it can be found that the higher the probability value, the lower the energy value and the more stable.

### Identification of the critical gene in transition

Studying the genes that play a crucial role in the transition is a meaningful task. In this section, we propose two indicators for identifying critical genes in the transition and determining whether they have a crucial role in promoting or inhibiting the transition.

#### Indicator based on transition probability

In the aforementioned context, we utilize a random walk model to model the dynamical system and construct the identification of attractors and their transition probabilities in a data-driven manner. For the transition process from state A to state B, we aim to investigate the probability contribution of each gene to this transition. We employ the method of simulating gene knockout to study the impact of each gene on the transition probability, defined as follows:

9 $$\Delta {P}_{i}={P}_{{all}}^{A\to B}-{P}_{{{all}/}{\left\{{g}_{i}\right\}}}^{A\to B}$$

Where ${P}_{{all}}^{A\to B}$ represents the transition probability from state *A* to state *B*, which includes all gene expression information. ${P}_{{all}/\{{g}_{i}\}}^{A\to B}$ represents the transition probability from state *A* to state *B* after removing the gene expression information of gene ${g}_{i}$ and reconstructing the transition process. It can be observed that if $\varDelta {P}_{i} > 0$, it indicates that gene ${g}_{i}$ increases the transition probability, promoting the transition from state *A* to state *B*. Conversely, if $\varDelta {P}_{i} < 0$, it inhibits the transition.

#### Indicator based on barrier height

We construct a data-driven approach to identify attractors and their energy landscape. For the transition from state *A* to state *B*, the barrier height is defined as the energy difference between saddle point and the departing attractor (Supplementary Figure 1). We develop a simulated gene knockout method to investigate the impact of each gene on the barrier height, defined as follows,

10 $$\Delta {H}_{i}=-\left({H}_{{all}}^{A\to B}-{H}_{{{all}/}{\left\{{g}_{i}\right\}}}^{A\to B}\right)$$

The barrier height for the transition process from state *A* to state *B* in the energy landscape, which includes all gene expression information, is denoted as ${H}_{{all}}^{A\to B}$. The barrier height in the energy landscape reconstructed after removing the gene expression information of gene ${g}_{i}$ is denoted as ${H}_{{all}/\{{g}_{i}\}}^{A\to B}$. It can be observed that if $\varDelta {H}_{i} > 0$, it indicates that gene ${g}_{i}$ reduces the barrier height, promoting the transition from state *A* to state *B*. Conversely, if $\varDelta {H}_{i} < 0$, it inhibits the transition. Compared with the definition of the change in transition probability, a negative sign has been added in order to keep it greater than 0 for indicating a promote effect.

### Data processing and functional analysis

The kidney renal clear cell carcinoma (KIRC) data was obtained from The Cancer Genome Atlas (TCGA) database (GDC (cancer.gov), specifically the RNA-seq data from tumor and tumor-adjacent samples, along with the corresponding clinical information. The tumor samples were then classified into different stages based on the available clinical information, obtained from TCGA. Samples without stage information were excluded from the analysis. The full clinical staging information can be found in Supplementary Table 1. The downloaded data consists of RNA sequencing data in Fragments Per Kilobase of exon model per Million mapped fragments (FPKM) format. The raw data was cleaned and analyzed for differential expression (Supplementary Figure 6). We also proposed approaches to mitigate any potential bias in the subsequent calculations. Firstly, we need to ensure an adequate sample size, for example, exceeding 300 samples in total, and at least 50 samples per stage, to ensure a certain statistical power in the calculations. Secondly, in the preprocessing stage, outliers can be removed or their impact on the calculations can be mitigated through standardization.

In the R environment, we performed differential expressed gene (DEG) analysis on the raw gene expression data using the DESeq2 package. The analysis results for all genes have been added to the Supplementary files. The Scanpy Python package was utilized for various analyses, including differential expression analysis and dimensionality reduction clustering^52^. KEGG Mapper tool (KEGG Mapper Color) and the DAVID Functional Annotation Tool (DAVID: Functional Annotation Tools (ncifcrf.gov) were used to perform the enrichment analysis.

### Reporting summary

Further information on research design is available in the Nature Research Reporting Summary linked to this article.

### Supplementary information

Supplementary Information

REPORTING SUMMARY

## Data Availability

Kidney renal clear cell carcinoma (KIRC), is available from the cancer genome atlas (TCGA) database (http://cancergenome.nih.gov). The computational codes are publicly available on GitHub (https://github.com/liujuntan/Landscape_KIRC).

## References

[1] McSharry PE, Smith LA, Tarassenko L (2003). Prediction of epileptic seizures: are nonlinear methods relevant?. Nat. Med..

[2] Sardanyés J (2015). Activation of effector immune cells promotes tumor stochastic extinction: A homotopy analysis approach. Appl Math. Comput..

[3] Itik M, Salamci MU, Banks SP (2009). Optimal control of drug therapy in cancer treatment. Nonlinear Anal.: Theory, Methods Appl..

[4] Li C, Wang J (2014). Quantifying the underlying landscape and paths of cancer. J. R. Soc. Interface.

[5] Bocci F, Zhou P, Nie Q (2022). spliceJAC: transition genes and state-specific gene regulation from single-cell transcriptome data. Mol. Syst. Biol..

[6] Sha Y, Wang S, Zhou P, Nie Q (2020). Inference and multiscale model of epithelial-to-mesenchymal transition via single-cell transcriptomic data. Nucleic Acids Res..

[7] Brabletz T, Kalluri R, Nieto MA, Weinberg RA (2018). EMT in cancer. Nat. Rev. Cancer.

[8] Kalluri R (2009). EMT: when epithelial cells decide to become mesenchymal-like cells. J. Clin. Investig..

[9] Li C, Balazsi G (2018). A landscape view on the interplay between EMT and cancer metastasis. NPJ Syst. Biol. Appl..

[10] Bray F, Laversanne M, Weiderpass E, Soerjomataram I (2021). The ever-increasing importance of cancer as a leading cause of premature death worldwide. Cancer.

[11] The global challenge of cancer. *Nat. Cancer* **1**, 1–2 (2020). https://www.nature.com/articles/s43018-019-0023-9#citeas. 10.1038/s43018-019-0023-9 35121840

[12] Dama E (2021). Biomarkers and lung cancer early detection: State of the art. Cancers.

[13] Debela DT (2021). New approaches and procedures for cancer treatment: Current perspectives. SAGE Open Med..

[14] Eusebi LH, Telese A, Marasco G, Bazzoli F, Zagari RM (2020). Gastric cancer prevention strategies: A global perspective. J. Gastroenterol. Hepatol..

[15] Berk Ş, Kaya S, Akkol EK, Bardakçı H (2022). A comprehensive and current review on the role of flavonoids in lung cancer–Experimental and theoretical approaches. Phytomedicine.

[16] Kang X, Li C (2021). A dimension reduction approach for energy landscape: identifying intermediate states in metabolism-EMT network. Adv. Sci..

[17] Ye L, Feng J, Li C (2023). Controlling brain dynamics: Landscape and transition path for working memory. PLoS Comput. Biol..

[18] Zhou P, Wang S, Li T, Nie Q (2021). Dissecting transition cells from single-cell transcriptome data through multiscale stochastic dynamics. Nat. Commun..

[19] Buczek M, Escudier B, Bartnik E, Szczylik C, Czarnecka A (2014). Resistance to tyrosine kinase inhibitors in clear cell renal cell carcinoma: from the patient’s bed to molecular mechanisms. Biochimica et. Biophysica Acta (BBA)-Rev. Cancer.

[20] Gulati S (2014). Systematic evaluation of the prognostic impact and intratumour heterogeneity of clear cell renal cell carcinoma biomarkers. Eur. Urol..

[21] Papa I, Vinuesa CG (2018). Synaptic interactions in germinal centers. Front. Immunol..

[22] Zhang H, Kong Q, Wang J, Jiang Y, Hua H (2020). Complex roles of cAMP–PKA–CREB signaling in cancer. Exp. Hematol. Oncol..

[23] Tanikawa C (2017). The transcriptional landscape of p53 signalling pathway. EBioMedicine.

[24] Xiao D (2020). Comparative gene expression analysis in melanocytes driven by tumor cell-derived exosomes. Exp. Cell Res..

[25] Escobar-Hoyos LF (2014). Keratin 17 in premalignant and malignant squamous lesions of the cervix: proteomic discovery and immunohistochemical validation as a diagnostic and prognostic biomarker. Mod. Pathol..

[26] McCawley LJ, Wright J, LaFleur BJ, Crawford HC, Matrisian LM (2008). Keratinocyte expression of MMP3 enhances differentiation and prevents tumor establishment. Am. J. Pathol..

[27] Martinelli CMD (2017). MGMT and CALCA promoter methylation are associated with poor prognosis in testicular germ cell tumor patients. Oncotarget.

[28] Lam KK (2021). The orphan nuclear receptor NR0B2 could be a novel susceptibility locus associated with microsatellite-stable, APC mutation-negative early-onset colorectal carcinomas with metabolic manifestation. Genes Chromosomes Cancer.

[29] Liu J, Ding D, Zhong J, Liu R (2022). Identifying the critical states and dynamic network biomarkers of cancers based on network entropy. J. Transl. Med..

[30] Liu R, Aihara K, Chen L (2013). Dynamical network biomarkers for identifying critical transitions and their driving networks of biologic processes. Quant. Biol..

[31] Liu R, Chen P, Chen L (2020). Single-sample landscape entropy reveals the imminent phase transition during disease progression. Bioinformatics.

[32] Lang J, Nie Q, Li C (2021). Landscape and kinetic path quantify critical transitions in epithelial-mesenchymal transition. Biophys. J..

[33] Sarkar S, Sinha SK, Levine H, Jolly MK, Dutta PS (2019). Anticipating critical transitions in epithelial–hybrid-mesenchymal cell-fate determination. Proc. Natl Acad. Sci..

[34] Luo RR (2020). Clostridium perfringens beta2 toxin induced in vitro oxidative damage and its toxic assessment in porcine small intestinal epithelial cell lines. Gene.

[35] Ganapathi MK (2016). Expression profile of COL2A1 and the pseudogene SLC6A10P predicts tumor recurrence in high-grade serous ovarian cancer. Int. J. Cancer.

[36] Gelman IH (2010). Emerging roles for SSeCKS/Gravin/AKAP12 in the control of cell proliferation, cancer malignancy, and barriergenesis. Genes Cancer.

[37] Choromańska A (2021). Modifications of plasma membrane organization in cancer cells for targeted therapy. Molecules.

[38] Hecht SS (2006). Cigarette smoking: cancer risks, carcinogens, and mechanisms. Langenbeck’s Arch. Surg..

[39] Staaf J (2020). Diagnostic Value of Insulinoma-Associated Protein 1 (INSM1) and comparison with established neuroendocrine markers in pulmonary cancers a comprehensive study and review of the literature. Arch. Pathol. Lab. Med..

[40] Patel RS (2022). A GATA4-regulated secretory program suppresses tumors through recruitment of cytotoxic CD8 T cells. Nat. Commun..

[41] Moon C. S., Moon D., & Kang S. K. Aquaporins in cancer biology. *Front. Oncol.* **12**, 782829 (2022). 10.3389/fonc.2022.782829 PMC9278817 35847914

[42] Vivero M, Doyle LA, Fletcher CDM, Mertens F, Hornick JL (2014). GRIA2 is a novel diagnostic marker for solitary fibrous tumour identified through gene expression profiling. Histopathology.

[43] Chen F, Li C (2022). Inferring structural and dynamical properties of gene networks from data with deep learning. NAR Genom. Bioinform..

[44] Vanden-Eijnden E (2010). Transition-path theory and path-finding algorithms for the study of rare events. Annu. Rev. Phys. Chem..

[45] Vanden-Eijnden E (2006). Towards a theory of transition paths. J. Stat. Phys..

[46] Metzner P, Schütte C, Vanden-Eijnden E (2009). Transition path theory for Markov jump processes. Multiscale Model. Simul..

[47] Bowman G. R., Pande V. S., & Noé F. *An Introduction to Markov State Models and Their Application to Long Timescale Molecular Simulation* (Springer Science & Business Media, 2013, vol. 797).

[48] Wolf FA (2019). PAGA: graph abstraction reconciles clustering with trajectory inference through a topology preserving map of single cells. Genome Biol..

[49] Haghverdi L, Büttner M, Wolf FA, Buettner F, Theis FJ (2016). Diffusion pseudotime robustly reconstructs lineage branching. Nat. Methods.

[50] Street K (2018). Slingshot: cell lineage and pseudotime inference for single-cell transcriptomics. BMC Genomics.

[51] Qiu X (2017). Reversed graph embedding resolves complex single-cell trajectories. Nat. Methods.

[52] Wolf FA, Angerer P, Theis FJ (2018). SCANPY: large-scale single-cell gene expression data analysis. Genome Biol..

