# Supplementary material for: Data-driven energy landscape reveals critical genes in cancer progression
Source: NPJ Syst Biol Appl. 2024 Mar 8;10:27. doi: 10.1038/s41540-024-00354-4 (PMC10923824; doi:10.1038/s41540-024-00354-4)
Supplement: Supplementary file 1 — Supplementary Information [file 41540_2024_354_MOESM1_ESM.pdf]

Supplementary Information for

Data-driven energy landscape reveals critical genes in cancer progression

Juntan Liu, Chunhe Li

Chunhe Li.

E-mail: chunheli@fudan.edu.cn

This PDF file includes:

Supplementary Notes

Supplementary Tables 1 to 2

Supplementary Figures 1 to 6

Supplementary References

Supplementary Note 1: The choice of attractor numbers

The determination of cluster numbers remains a challenging problem in single-cell transcriptome data analysis. In MuTrans implementation, the following strategy is used to determine the number of attractors:

Given the constructed cell-cell random walk transition probability matrix (rwTPM), we can calculate the eigenvalues and use the eigen-peak index (EPI) to provide the reference of attractors number. Suppose that we have the eigenvalues of symmetric cell-cell weight matrix $W(x,y)$, we then sort the eigenvalues of normalized weight matrix $D^{-\frac{1}{2}}WD^{-\frac{1}{2}}$ as $|\lambda_1|>|\lambda_2|>...>|\lambda_N|$. The *kth* EPI is defined as $\frac{\lambda_k^2}{\lambda_{k+1}^2}$, when a peak of the EPI is observed at index *k*, we reason that there is an obvious eigen-gap in the normalized graph Laplacian of the cellular network, and therefore *k* is a candidate for the number of clusters. Note that we may observe multiple peaks, which means that we may choose different *k* on resolutions.

We also combine with prior biological knowledge, marker genes analysis, or compare with labels in original publications to further validate the results.

Supplementary Note 2: Theoretical background

Cellular evolution can often be modeled as a dynamical system using stochastic differential equations (SDEs), as follows：

$$dX_t = f(X_t)dt + \sigma(X_t)dW_t \tag{1}$$

where $\boldsymbol{X}_t \in \mathbb{R}^p$ is the gene expression value of the cell at the moment t, $f(X_t)$ is the driving force containing the interaction relationship between genes, etc., W_t is the standard Brownian motion accounting for the noise in gene expression dynamics, and $\sigma(X_t)$ is the noise amplitude of the system. When the number of genes is small, $f(X_t)$ can be estimated using causal inference algorithms[1]. However, when the number of genes is too large (typically exceeding the limit in single-cell sequencing), direct fitting or solving of high-dimensional equations (1) is not feasible. In such cases, a multiscale data-driven method is employed to reconstruct the structure of the dynamical system, where each steady state of the system is represented as an attractor. This approach is further described in the following section.

Supplementary Note 3: The workflow of the MuTrans algorithm

The Mutrans algorithm aims to reveal the dynamics inherent in data by addressing three aspects[2]:

- Computation of the random walk transition probability matrix (rwTPM) at the cell-cell level.
- Identification of attractors in the nonlinear dynamical system, classification of each cell accordingly, and computation of the rwTPM at the cluster-cluster level. Subsequently, a lineage inference approach can be employed to deduce the transition paths between attractors.
- Computation of the rwTPM at the cell-cluster level. Utilizing membership probability allows for the representation of the cell's likelihood of belonging to the attractor.

By encompassing these perspectives, the Mutrans algorithm offers valuable insights into the dynamics of data. A multi-scale analysis of the random walk transition probability matrix (rwTPM) is performed as follows.

Computing the rwTPM at the cell-cell level

The transition probability matrix, measured at the cell-cell level using the random walk model, can be directly calculated from the gene expression data. It is defined as follows:

$$p(x,y)=\frac{w(x,y)}{d(x)},d(x)=\sum_{z}w(x,z) \tag{2}$$

In this context, x,y represents the cell, and $w(x,y)$ represents the distance between cells x and y. The distance metric used can be Euclidean distance, cosine similarity, correlation coefficient, or any other appropriate measure. Under this definition, the stationary probability distribution of the transition probability matrix is denoted as

$\mu(x)=\frac{d(x)}{\sum_z d(z)}$, and it satisfies the detailed-balance condition $\mu(x)p(x,y)=\mu(y)p(y,x)$.

Computing the rwTPM at the cluster-cluster level

In this step, the number of attractors needs to be determined. This can be achieved by employing the EPI strategy (Supplementary Note 1; Supplementary Figure 2) to assess the gene expression data. Alternatively, if the label information of cells or marker genes of cells is known, prior knowledge about the number of label categories can be used to determine the number of attractors.

The transition probability matrix $\overline{\mathrm{P}}=(\overline{P}_{ij})_{K\times K}$ is first defined at the cluster-cluster level, where $\overline{P}_{ij}$ represents the probability of attractor S_i transiting to S_j, K represents the number of attractors, and based on the cluster-cluster random walk transition probability matrix (rwTPM) $\overline{\mathrm{P}}=(\overline{P}_{ij})_{K\times K}$, the cell-cell rwTPM is further constructed as follows:

$$\overline{p}(x,y)=\sum_{i,j}1_{S_i}(x)\overline{P}_{ij}1_{S_j}(y)\frac{\mu(y)}{\overline{\mu}_j} \quad (3)$$

where $\overline{\mu}_j=\sum_y 1_{S_j}(y)\mu(y)$, and $1_{S_j}(z)$ is the indicator function, which means that if the cell z belongs to attractor S_j, then $1_{S_j}(z)=1$, otherwise $1_{S_j}(z)=0$.

For cluster-cluster rwTPM $\overline{\mathrm{P}}=(\overline{P}_{ij})_{K\times K}$ and the attractor clustering result, we can calculate them using the optimization method as follows:

$$\min_{S_K,\overline{P}_{ij}}\left\|\overline{p}[S_K,\overline{P}_{ij}]-p\right\|_\mu^2 \quad (4)$$

where μ is the stationary probability distribution of the cell-cell rwTPM p, $\|A\|_\mu^2=\sum_{x,y}\frac{\mu(x)}{\mu(y)}A(x,y)^2$, and this optimization problem can be solved iteratively[2]. The optimized $S_K{}^*,\overline{P}_{ij}^*$ obtained in this study measure the stability of the system and its

inter-transition characteristics using a probabilistic model. Additionally, the Most Probable Path Tree (MPPT) approach or Maximum Probability Flow Tree (MPFT) approach can be applied to infer the transition paths between attractors.

Computing the rwTPM at the cell-cluster level

Constructing cell-cluster rwTPM by introducing membership function $q(x) = (q_1(x), q_2(x), .., q_K(x))$, where $q_i(x)$ represents the probability that cell x belongs to attractor S_i, and $\sum_i q_i(x) = 1$. Based on $\overline{\mathrm{P}} = (\overline{P}_{ij})_{K\times K}$ obtained from the previous step, the cell-cell rwTPM can be constructed as follows:

$$\tilde{p}(x, y) = \sum_{i,j} q_i(x)\overline{P}_{ij}q_j(y)\frac{\mu(y)}{\tilde{\mu}_j}, \tilde{\mu}_j = \sum_x q_j(x)\mu(x) \tag{5}$$

The solution for $q(x)$ can be obtained by the following optimization problem,

$$\min_q \|\tilde{p}[q] - p\|_\mu^2 \tag{6}$$

$$s.t. q(x) = (q_1(x), ..., q_K(x)), \sum_{i=1}^{K} q_i(x) = 1$$

where $\overline{\mathrm{P}} = (\overline{P}_{ij})_{K\times K}$ is optimized when constructing the cluster-cluster rwTPM, this problem can be solved by the quasi-Newton method[2].

Supplementary Table 1: The number of tumor samples within each stage in the KIRC cancer dataset from TCGA.

	**TA**	**Stage I**	**Stage II**	**Stage III**	**Stage IV**
**KIRC**	73	197	41	112	68

TA samples: tumor-adjacent samples

Supplementary Table 2: The top ten promoting genes detected by transition probability indicator or barrier height indicator in the process from stage TA to I. *Log2FC* represents the log2-fold change in gene expression levels between different conditions, and *P_adj* represents the statistically significant level after multiple testing correction.

**Gene**	ΔP	ΔH	***Log2FC***	***P_adj***
AQP10	-1.871	0.029	0.850	0.004323066
ATP12A	1.178	0.074	6.386	2.44E-242
CALCA	2.078	-0.087	3.524	3.39E-39
CNTN5	0.283	0.040	1.106	2.01E-07
**CPB2**	-1.868	0.041	-0.164	**0.54924432**
FXYD4	2.272	-0.099	4.452	1.07E-64
GABRA2	1.228	0.995	4.678	5.84E-98
GCGR	1.188	-0.075	4.416	1.04E-63
GCKR	0.010	1.022	-2.376	6.52E-17
INHBE	0.201	-0.011	-2.574	5.34E-21
IRF6	0.316	-0.011	1.923	2.85E-30
**KRT4**	0.295	0.033	-0.222	**0.377797491**
**MC4R**	0.101	0.035	-0.006	**0.981625535**
PIK3C2G	-0.057	2.741	6.914	0
PLA2G4F	1.138	0.022	4.951	1.45E-92
PPP1R1B	-0.570	0.073	4.355	2.01E-60

Genes marked in bold format indicate non-differentially expressed genes (*p_adj*> 0.05). The transition probability indicator is defined as $\Delta P_i = P_{all}^{A\to B} - P_{all/\{g_i\}}^{A\to B}$, where $P_{all}^{A\to B}$ and $P_{all/\{g_i\}}^{A\to B}$ represent the transition probability from state *A* to state *B* before and after the gene g_i knockout respectively. The barrier height indicator is defined as $\Delta H_i = -(H_{all}^{A\to B} - H_{all/\{g_i\}}^{A\to B})$, where $H_{all}^{A\to B}$ and $H_{all/\{g_i\}}^{A\to B}$ represent the barrier height from state A to state B before and after the gene g_i knockout respectively.

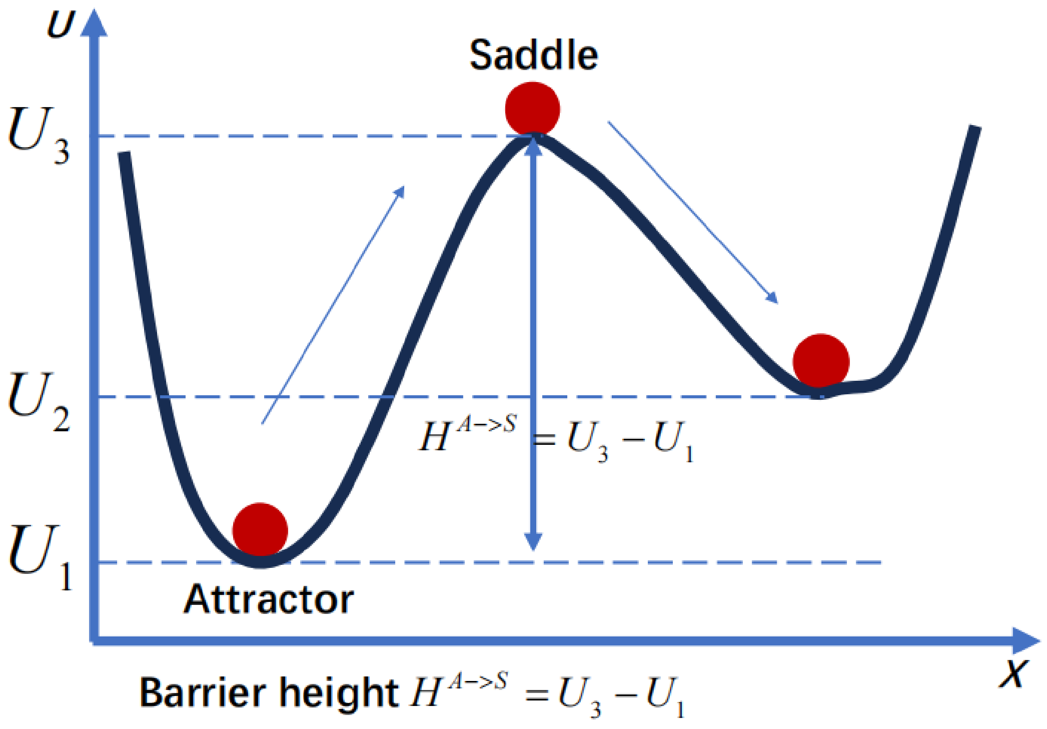

Supplementary Figure 1: Visualization of the definition of barrier height.

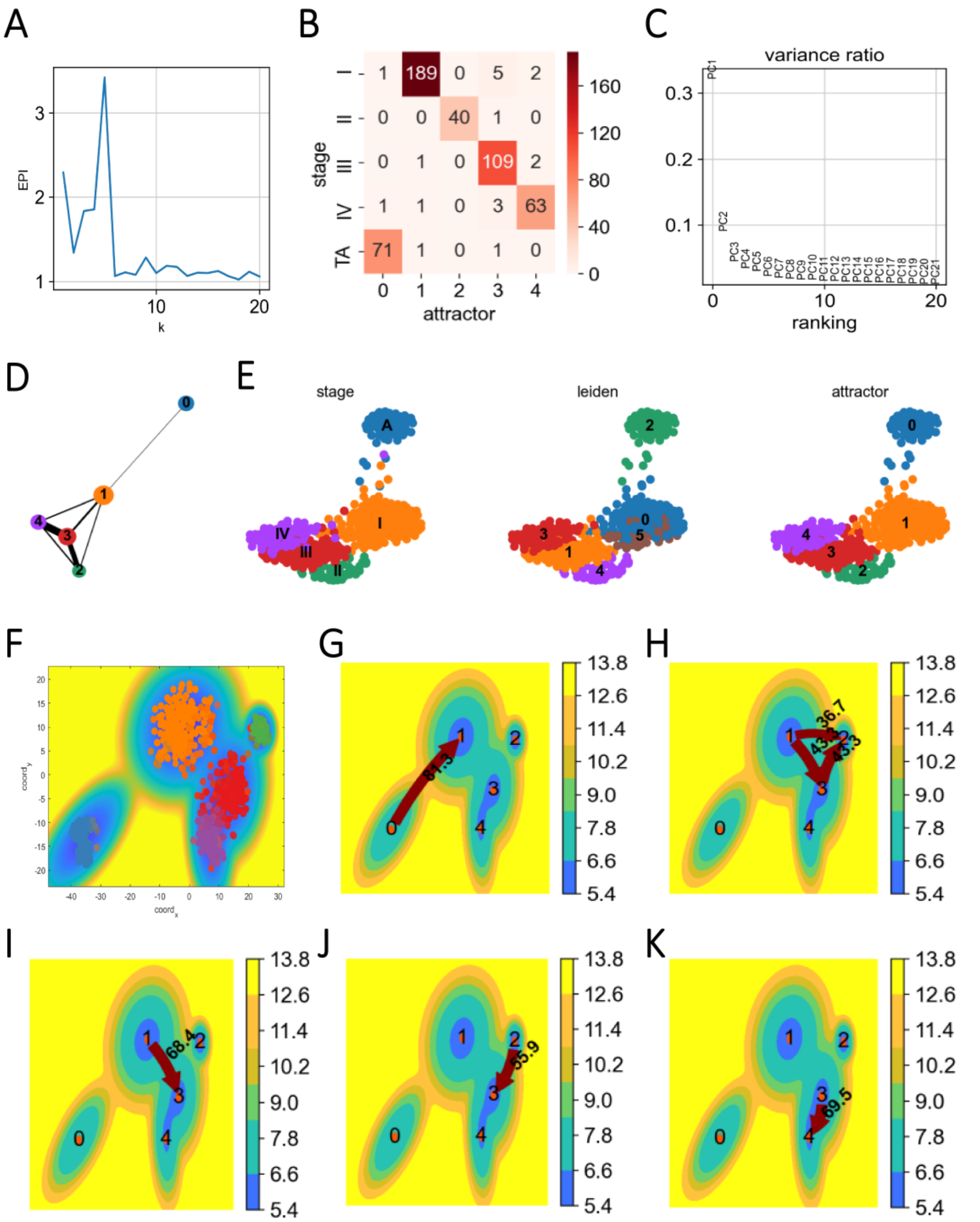

Supplementary Figure 2: (A)EPI indicator: it shown a peak in *k*=5, so the number of attractors is set as 5. **(B) number of stage by attractor:** we can found that each stage can be modeled as an attractor. **(C) PCA analysis:** it need the principal component analysis (PCA) result when compute t-SNE algorithm, and we can see the top 10 PCAs can represents the most information. **(D) PAGA analysis:** we can see the transition path was consistent with the MPFT and MPPT. **(E) samples plot by label in the PAGA plane. (F) two-dimension energy landscape.** Transition probability in the process (G)attractor 0->1 (H)attractor 1->2 (I)attractor 1->3 (J)attractor 2->3 (K)attractor 3->4.

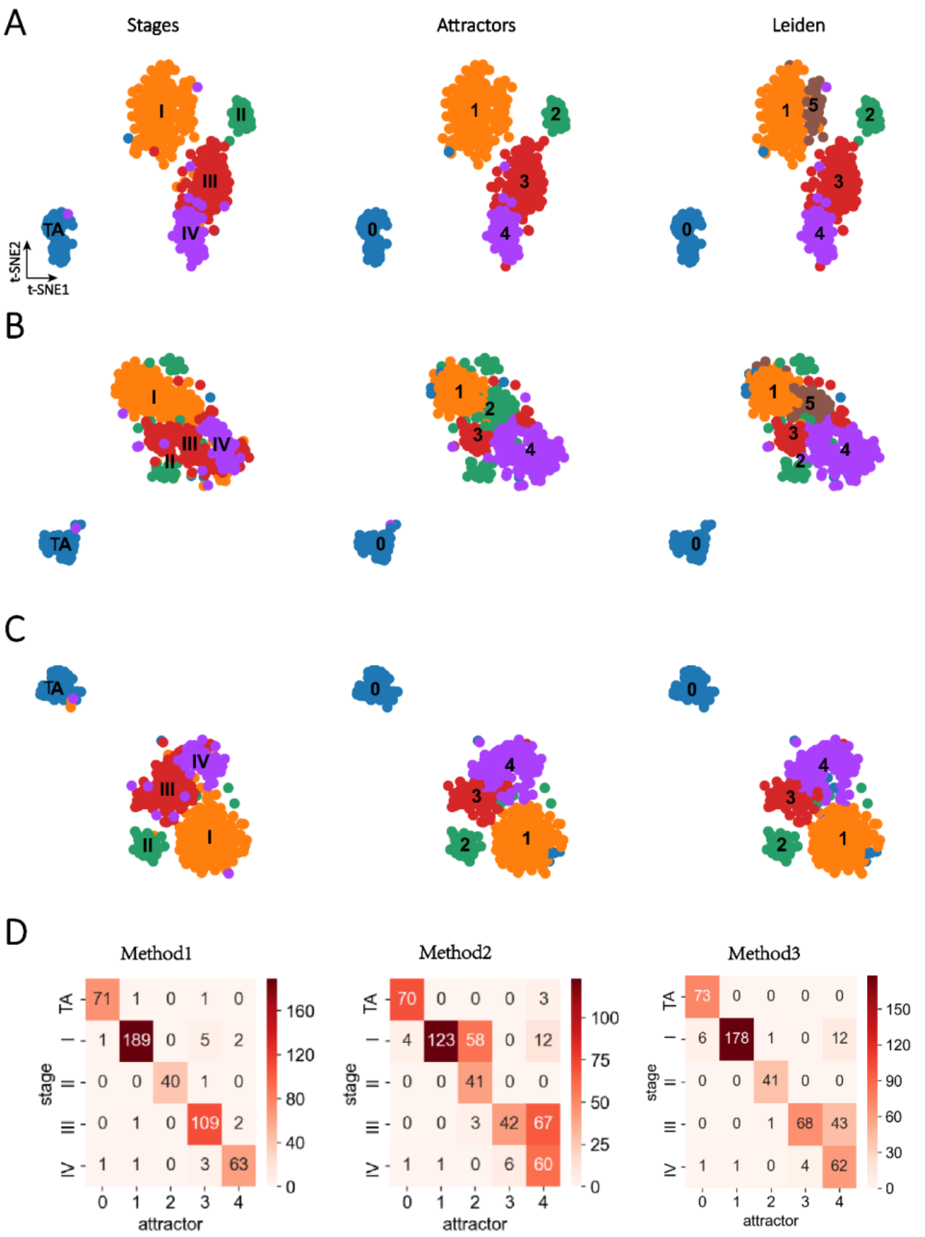

Supplementary Figure 3: The clustering results of three normalization methods. Method 1: taking the logarithm of the gene expression matrix (utilized in our original experiment). Method 2: scaling the data to unit variance and zero mean (achieved using Python function *scanpy.pp.scale()*). Method 3: scaling the per cell to unit variance and zero mean and then taking the logarithm of the normalized data (using Python function *scanpy.pp.recipe_zheng17()*). (A) The clustering result of attractors obtained by normalization method 1. (B) The clustering result of attractors obtained by normalization method 2. (C) The clustering result of attractors obtained by normalization method 3. (D) The correspondence between the result of attractors using methods 1-3 and the stage labels of the samples. It can be observed that method 1 almost uniquely associates each stage label with each attractor. In contrast, in method 2, the samples of stages I and III correspond to multiple attractors, indicating a less satisfactory correspondence. The same applies to method 3, where the samples of stage III do not completely correspond to attractor 3, and there are still a considerable number of samples corresponding to attractor 4.

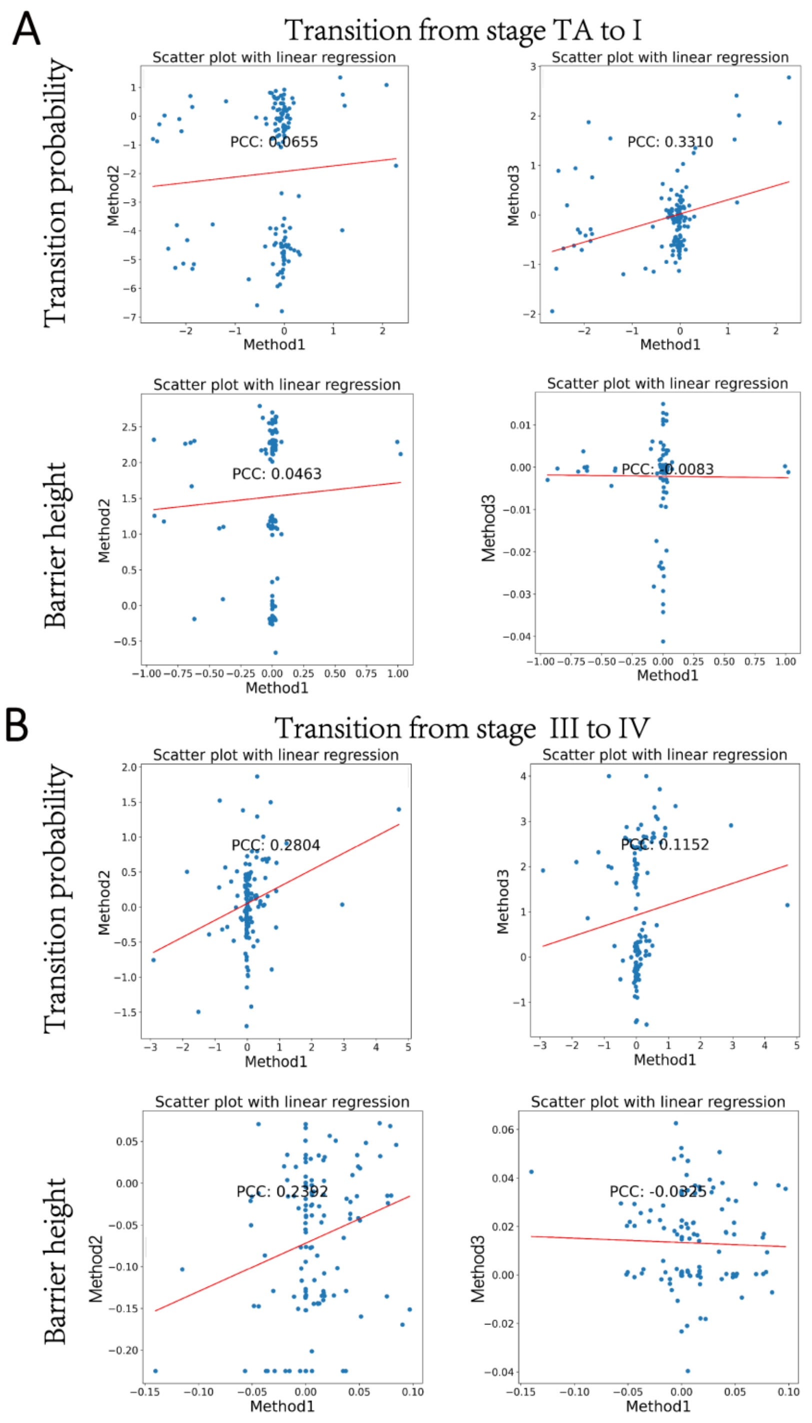

Supplementary Figure 4: The impact of three normalization methods on transition probability and barrier height. (A) Transition from stage TA to I. Each point in the figure represents the coordinate values of the transition probability (barrier height) of each gene under the two methods. It can be observed from the figure that only in the case of the transition probability indicator, methods 1 and 3 exhibit a strong linear relationship (Pearson correlation coefficient (PCC)=0.3310). (B) Transition from stage III to IV. Similar to Supplementary Figure 4A, each point represents the coordinate values of the transition probability (barrier height) of each gene under the two methods. It can be observed from the figure that methods 1 and 2, as well as methods 3, demonstrate a weak linear relationship.

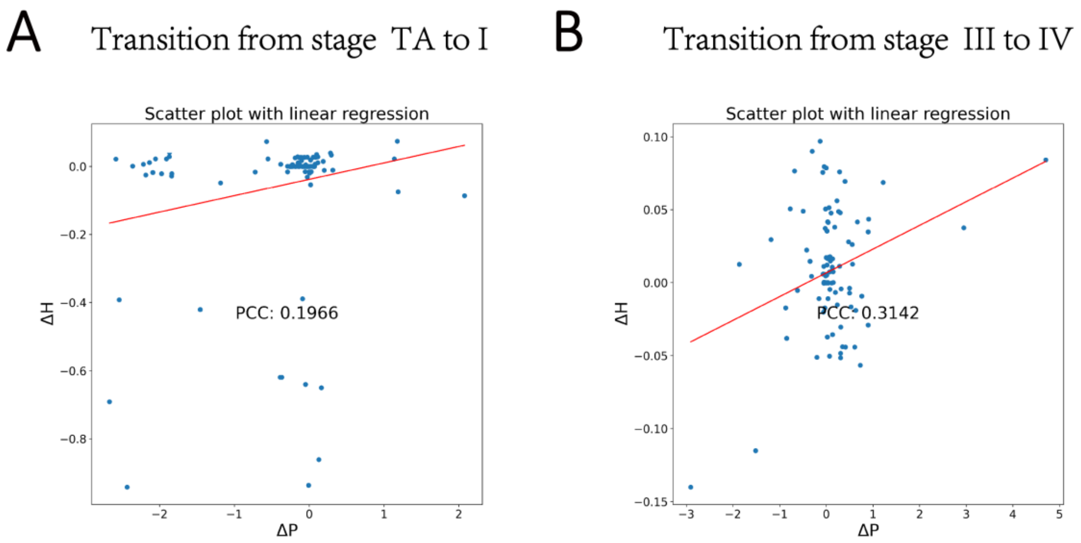

Supplementary Figure 5: Scatter plots and their linear regressions for two indicators, based on transition probability and barrier height. (A) In the transition from stage TA to I. Each point represents the value of the gene under the two indicators. Additionally, linear regression curves were fitted, along with the Pearson correlation coefficient (PCC) values of 0.1966. (B) In the transition from stage III to IV. Similarly, linear relationships were fitted for two indicators, with PCC=0.3142. We observed that both indicators exhibited a certain degree of linear relationship in the above two processes.

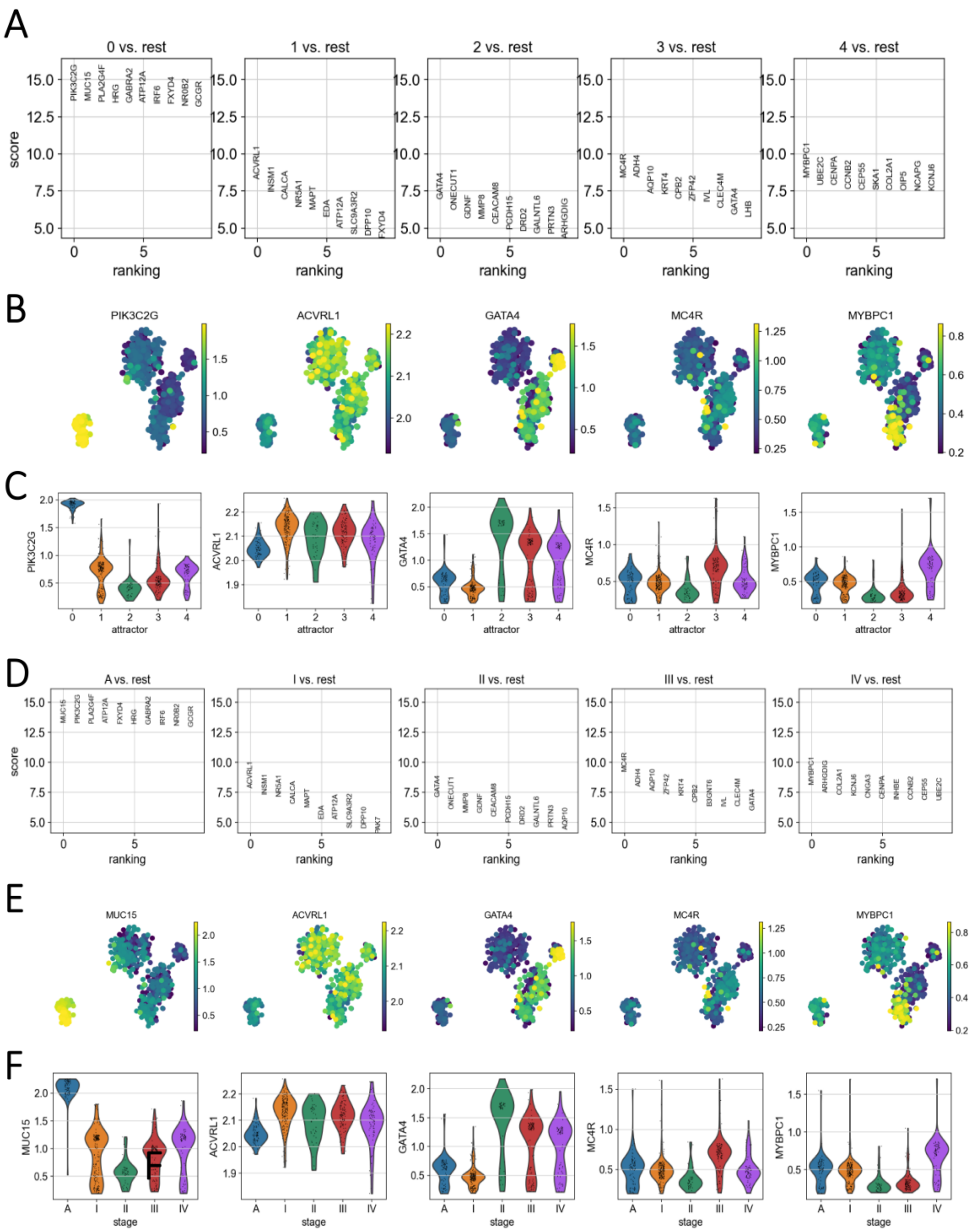

Supplementary Figure 6: The raw data was analyzed for differential expression. (A) DEG analysis by attractor. (B) The 1-st DEG (marker gene) in each attractor. (C) Boxplot of marker gene by attractor. (D) DEG analysis by stage. (E) The 1-st DEG (marker gene) in each stage. (F) Boxplot of marker gene by stage.

Supplementary references

1. Chen F, Li C: **Inferring structural and dynamical properties of gene networks from data with deep learning**. *NAR Genom Bioinform* 2022, **4**(3):lqac068.
2. Zhou P, Wang S, Li T, Nie Q: **Dissecting transition cells from single-cell transcriptome data through multiscale stochastic dynamics**. *Nat Commun* 2021, **12**(1):5609.
